# Delayed Diagnosis and Treatment of Rare Fungal Peritoneal Dialysis-Associated Peritonitis

**DOI:** 10.7759/cureus.66796

**Published:** 2024-08-13

**Authors:** Grace Wang, Lucas McKnight, Beth A Vogt

**Affiliations:** 1 Internal Medicine and Pediatrics, Western Michigan University, Kalamazoo, USA; 2 Internal Medicine and Pediatrics, Nationwide Children's Hospital and The Ohio State University Wexner Medical Center, Columbus, USA; 3 Pediatric Nephrology, Nationwide Children’s Hospital, Columbus, USA

**Keywords:** sepsis, kodamaea ohmeri, end-stage renal disease (esrd), patient education, provider education, delayed diagnosis, invasive fungal infections, peritoneal dialysis related peritonitis, peritoneal dialysis (pd)

## Abstract

Fungal peritonitis is a somewhat rare yet serious complication associated with peritoneal dialysis (PD). It requires prompt diagnosis and treatment to prevent unnecessary morbidity and mortality. We present an unusual presentation that highlights the consequences of delayed diagnosis and management and propose methods for improving care for patients receiving peritoneal dialysis.

## Introduction

Fungal peritonitis is a rare yet serious complication of peritoneal dialysis, associated with 20-30% morbidity and mortality [1-4]. As such, it requires prompt diagnosis and treatment. Most cases are caused by Candida species, which make up 80% or more of the total cases in pediatrics [1]. Also, the consequence is that fungal peritonitis is associated with a significant risk of technique failure through loss of peritoneal membrane function, with up to 40% of patients being unable to resume peritoneal dialysis (PD) due to peritoneal adhesions, sclerosis, and irreversible membrane damage [1,5]. We present a case of a teen with an unusual presentation and delayed diagnosis and treatment of PD-associated fungal peritonitis. We propose methods to promote early identification and treatment of peritonitis in patients receiving peritoneal dialysis, using patient, family, and provider education.

## Case presentation

**Figure 1:**
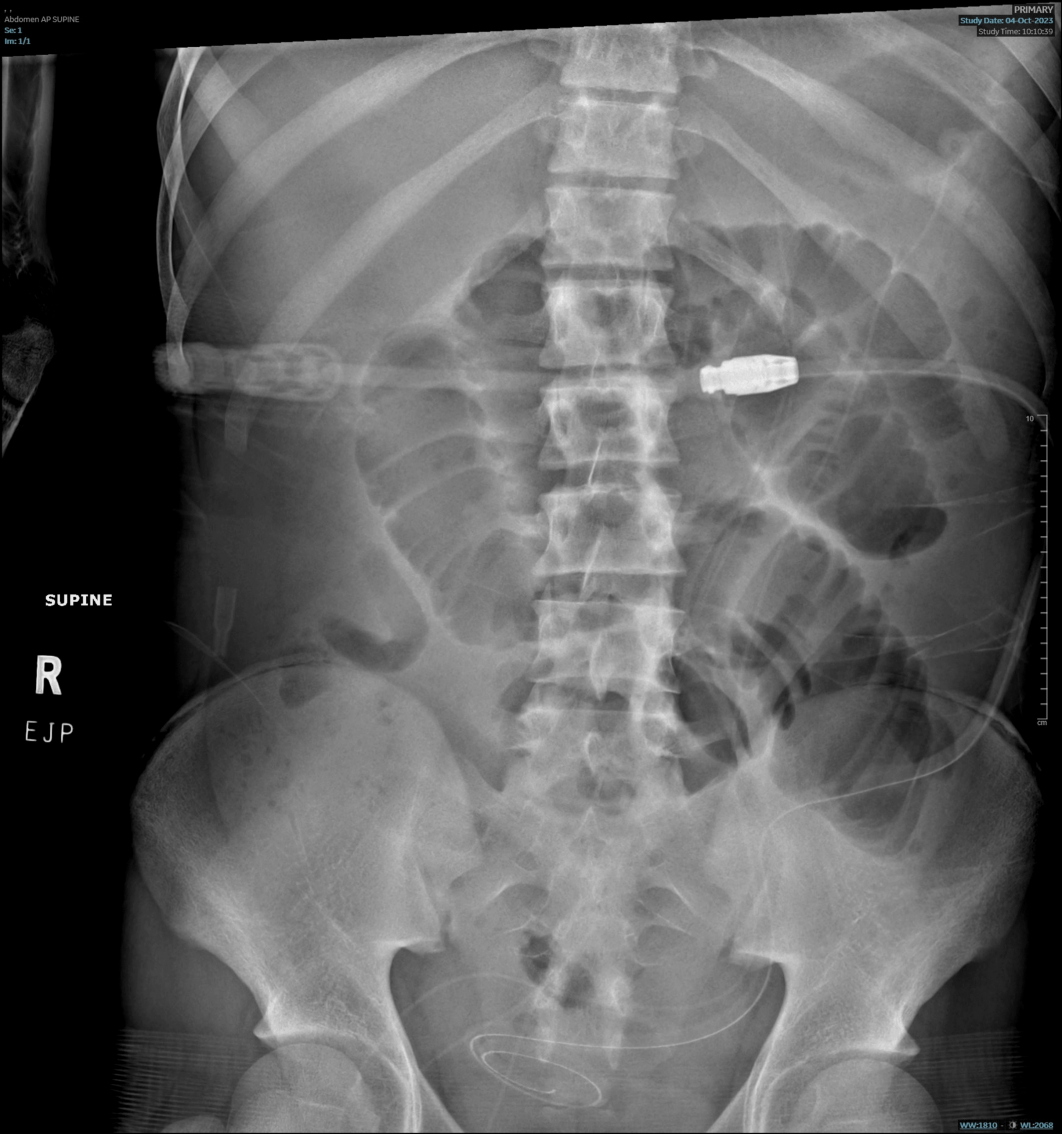
Abdominal x-ray showed distended centralized small bowel loops with air-fluid levels in a pattern typical for small bowel obstruction.

**Figure 2:**
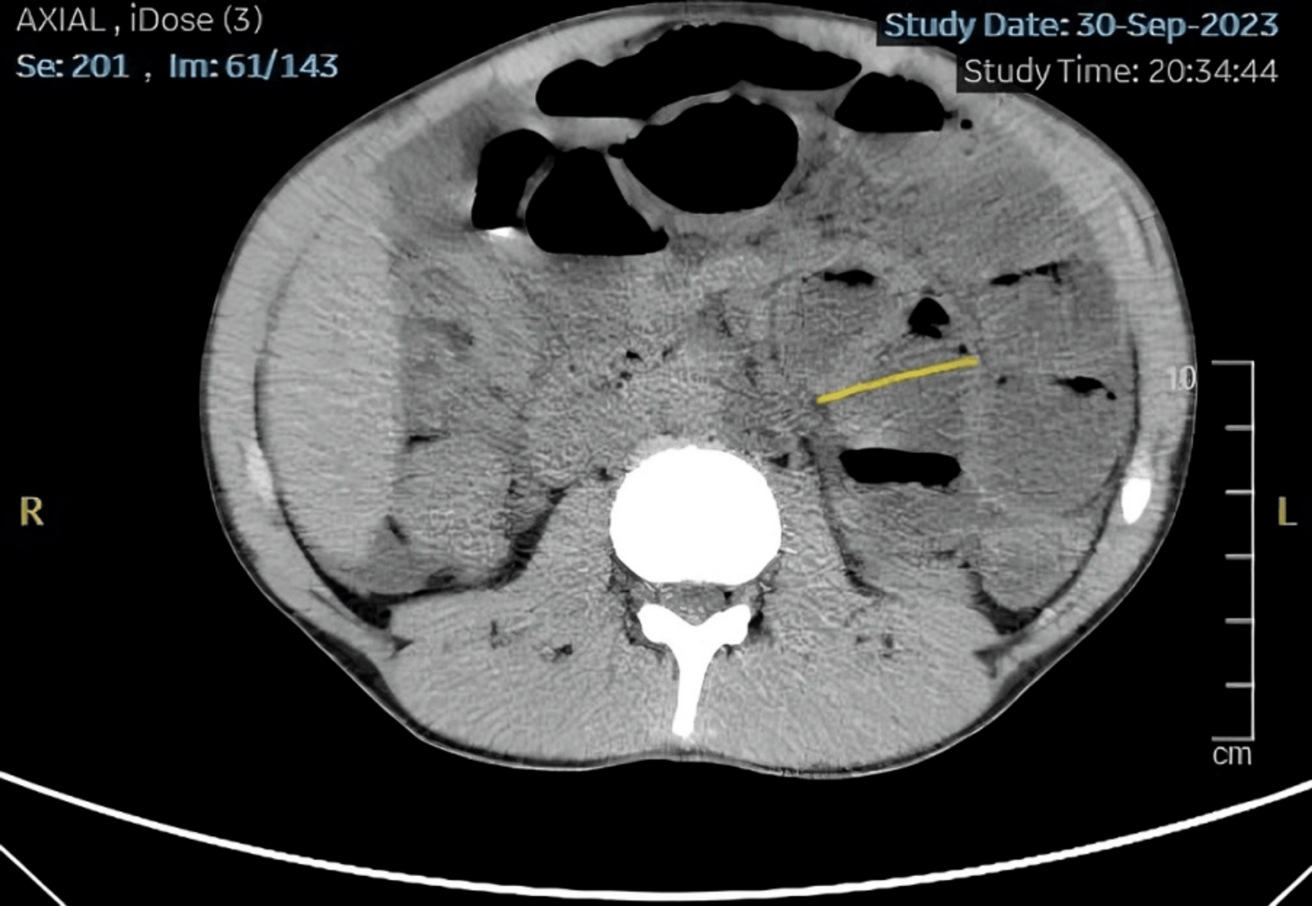
CT abdomen and pelvis. Dilated proximal small bowel loops in the left hemiabdomen (yellow line indicating dilatation up to 3.8 cm) with fluid-filled loops in the right hemiabdomen in a pattern consistent with small bowel obstruction.

A 16-year-old male with a history of end-stage renal disease due to IgA nephropathy on chronic peritoneal dialysis (PD) for eight months (surgically placed catheter) presented to our institution's emergency department with generalized abdominal pain, nausea, and vomiting and several days of fever with a max temperature of 102°F. Three days prior to presentation in our ED, he had presented to his local urgent care with similar symptoms and was diagnosed with viral gastroenteritis. Peritoneal effluent was not obtained at that time. When his symptoms worsened, he presented to an outside hospital ED a few days later, where an abdominal x-ray and subsequent CT scan showed findings suggestive of small bowel obstruction (SBO) (Figures 1, 2).

He was transferred to our institution, where he was found to be generally ill-appearing, tachycardic, febrile, hypoxic with oxygen saturation of ~80%, and endorsing generalized abdominal tenderness. He was fluid resuscitated and stabilized, placed on bowel rest, and a nasogastric (NG) tube was placed on recommendation of pediatric surgery for management of partial SBO. He was given IV ceftriaxone and IV metronidazole for broad-spectrum antimicrobial coverage. He was admitted to the pediatric nephrology service, where peritoneal effluent was ultimately obtained. The results showed a total cell count of >2063 WBC/µL with 49% neutrophils (PMNs), which is concerning for PD-associated peritonitis. He was started on intraperitoneal cefepime and IV fluconazole for fungal prophylaxis. Over the next 24 hours, his abdominal pain and vomiting improved, the NG tube was removed and his diet was slowly advanced. Peritoneal cell count improved to 1800 WBC/µL with 28% PMNs. Peritoneal effluent culture grew *Kodamaea ohmeri* (a yeast species), and he was started on IV amphotericin for a 14-day course. His PD catheter was urgently removed for source control. He was discharged with a tunneled hemodialysis catheter on 3x weekly hemodialysis with a plan for PD catheter replacement in several weeks. Although his peritoneal effluent cultures were negative for bacterial growth, he completed a 14-day course of ciprofloxacin and metronidazole, given that the cultures were obtained after antibiotic administration.

## Discussion

Literature suggests that *Kodamaea ohmeri *is a rare emerging yeast pathogen associated with high mortality rates. It has been found to cause various invasive infections including peritoneal catheter-associated peritonitis [6]. In a patient treated with PD presenting with generalized abdominal pain and fever, peritonitis should be a primary consideration in the differential diagnosis due to its risk for significant morbidity and mortality. Peritoneal effluent studies, including cell count, differential, Gram stain, and cultures (bacterial and, if suspicion, fungal), should be taken promptly when peritonitis is suspected, ideally before initiation of antibiotics to ensure an accurate identification of causative organisms [7]. For patients with PD, empiric intraperitoneal antibiotic therapy should include Gram-positive and Gram-negative coverage. Fungal prophylaxis is recommended whenever a PD patient is treated with antibiotics [8,9].

This patient presented to three healthcare settings with abdominal pain and fever, a clinical picture consistent with PD peritonitis. Peritoneal effluent was not obtained as part of any of these evaluations, likely related to front-line providers being less familiar with dialysis-associated complications. In addition, he and his family did not raise the possibility of PD-associated peritonitis to the providers, despite prior education about peritonitis risk. Ultimately, PD effluent studies obtained in the inpatient setting by his primary dialysis team confirmed a diagnosis of fungal peritonitis with cell count >100/µL, >50% polymorphonuclear leukocytes, and positive culture for *Kodamaea ohmeri*. Given the patient’s abdominal CT findings and oral intolerance, it is likely that he had a concomitant partial small bowel obstruction related to his infection.

Fungal peritonitis requires immediate catheter removal and at least two weeks of targeted antifungal therapy due to its high mortality and risk of biofilm formation [8,10]. Liposomal amphotericin B was selected to provide coverage for *Kodamaea ohmeri* due to the higher likelihood of resistance to other standard agents such as fluconazole. Although this patient’s peritoneal effluent did not grow any bacteria, he was treated with a full course of antibiotics due to the inability to rule out superimposed bacterial peritonitis given antibiotic pretreatment prior to culture collection.

## Conclusions

PD-associated fungal peritonitis has high morbidity and mortality and should be promptly diagnosed and treated. Early recognition and treatment can reduce the risk of permanent peritoneal membrane failure and inability to do PD. The possibility of peritonitis should be front of mind in PD patients presenting with generalized abdominal pain and fever. Peritoneal effluent fluid should be obtained as soon as peritonitis is suspected, followed by empiric antibiotic therapy, with appropriate antifungal prophylaxis. This case demonstrated a delay in diagnosis and initiation of appropriate treatment due to a lack of recognition of this dialysis-associated complication by multiple healthcare providers and families.

To mitigate delays in the diagnosis of peritonitis, patients and caregivers should feel empowered to communicate with their dialysis team and advocate for themselves in healthcare settings in cases of acute illness. In response to the gaps in care evident by this case, we propose methods to improve patient and provider awareness of PD complications including peritonitis. Interventions include the following: (1) providing families with educational handouts about peritonitis and strategies to advocate for their child’s care when providers are less familiar with PD complications; (2) educating ED staff about prompt recognition and management of peritonitis in PD patients; and (3) instituting a policy in our hospital that families should notify the dialysis team any time their child is seen for abdominal complaints and/or fever in an ED, urgent care, or primary care setting.

## References

[1] Matuszkiewicz-Rowinska J (2009). Update on fungal peritonitis and its treatment. Perit Dial Int.

[2] Juarez VD, Escobar KB, Ramirez ST, Quiroz IZ (2022). Fungal peritonitis associated with peritoneal dialysis due to non-albicans candida: a case series. Cureus.

[3] Shah PJ, Bergman S, Vegi S, Sundareshan V (2014). Fusarium peritonitis successfully managed with posaconazole and catheter removal. Perit Dial Int.

[4] Amici G, Grandesso S, Mottola A, Virga G, Calconi G, Bocci C (1994). Fungal peritonitis in peritoneal dialysis: critical review of six cases. Adv Perit Dial.

[5] Tapson JS, Mansy H, Freeman R, Wilkinson R (1986). The high morbidity of CAPD fungal peritonitis - description of 10 cases and review of treatment strategies. Q J Med.

[6] Zhou M, Li Y, Kudinha T, Xu Y, Liu Z (2021). Kodamaea ohmeri as an emerging human pathogen: a review and update. Front Microbiol.

[7] Perl J, Harel Z, Nessim SJ (2022). Peritoneal fluid analysis in peritoneal dialysis-associated peritonitis. JAMA.

[8] Li PK, Chow KM, Cho Y (2022). ISPD peritonitis guideline recommendations: 2022 update on prevention and treatment. Perit Dial Int.

[9] Restrepo C, Chacon J, Manjarres G (2010). Fungal peritonitis in peritoneal dialysis patients: successful prophylaxis with fluconazole, as demonstrated by prospective randomized control trial. Perit Dial Int.

[10] Prasad N, Gupta A (2005). Fungal peritonitis in peritoneal dialysis patients. Perit Dial Int.

